# Supplementary material for: Chromatin conformation regulates the coordination between DNA replication and transcription
Source: Nat Commun. 2018 Apr 23;9:1590. doi: 10.1038/s41467-018-03539-8 (PMC5913246; doi:10.1038/s41467-018-03539-8)
Supplement: Supplementary file 1 — Supplementary Information [file 41467_2018_3539_MOESM1_ESM.pdf]

Supplementary Information Almeida et al.

Chromatin conformation regulates the coordination between DNA replication and transcription

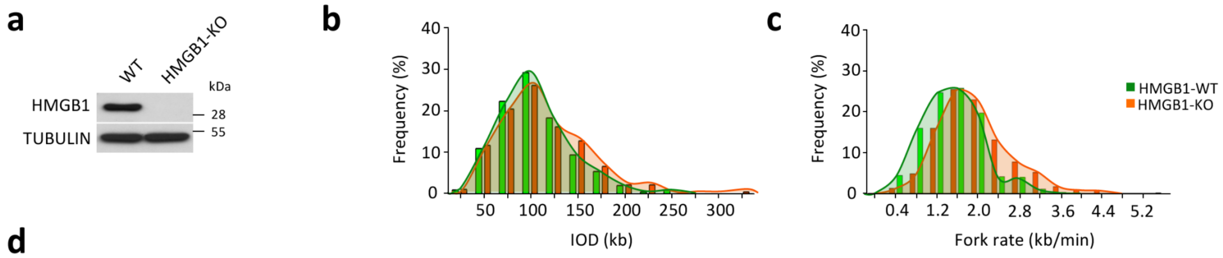

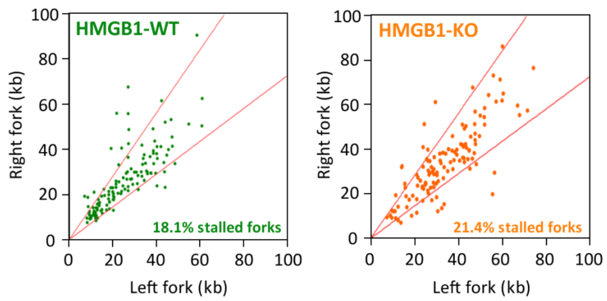

e

Figure 1f-h	IODs		Fork rate		Fork asymmetry	
	MEFs WT	MEFs HMGB1-KO	MEFs WT	MEFs HMGB1-KO	MEFs WT	MEFs HMGB1-KO
Number of values	202	180	627	596	133	126
Median	101.1	104.8	1.491	1.842	15.09	17.05
Mean	106.1	110.7	1.505	1.944	23.2	29.83
Std. deviation	39.92	43.87	0.5861	0.7341	28.16	40.4
P-value	-	0.3774	-	<0.0001	-	0.4398

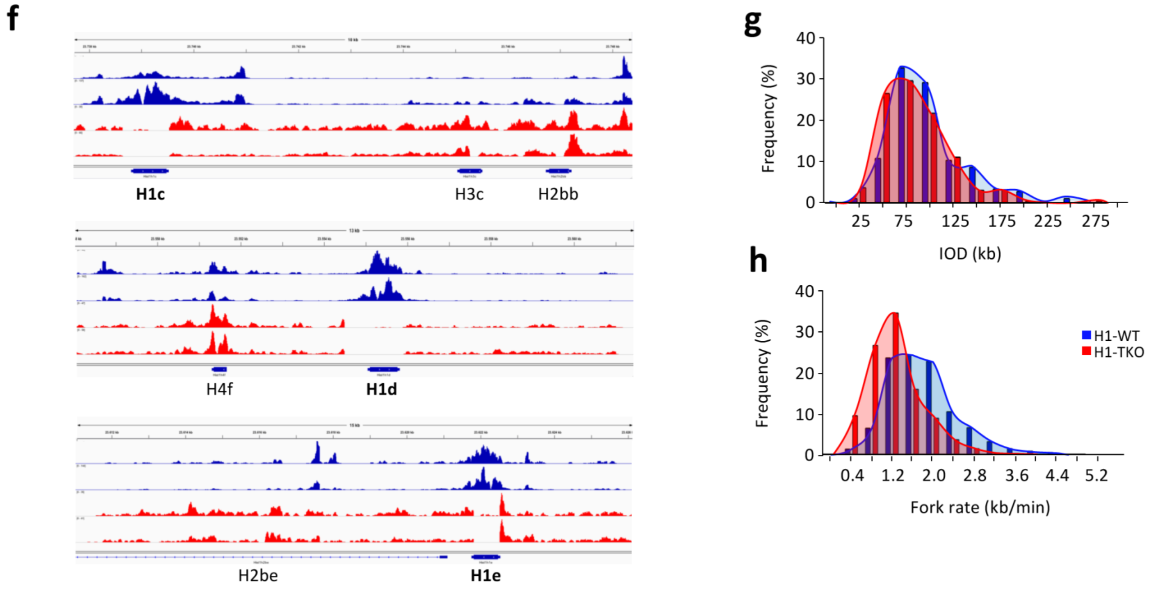

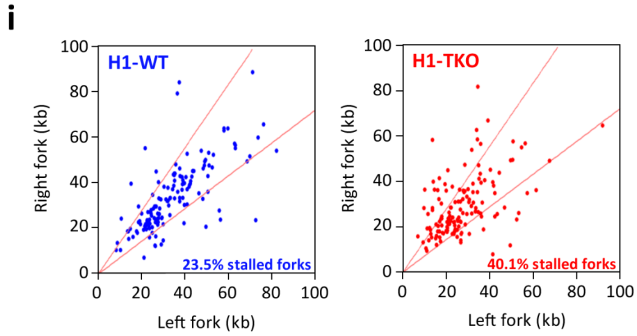

j

Figure 2b-d	IODs		Fork rate		Fork asymmetry	
	mES WT	mES H1-TKO	mES WT	mES H1-TKO	mES WT	mES H1-TKO
Number of values	175	189	679	684	140	144
Median	91.64	78.87	1.725	1.153	13.79	26.55
Mean	100.4	84.49	1.801	1.231	28.78	46.86
Std. deviation	38.86	35.63	0.6676	0.5234	39.93	63.15
P-value	-	<0.0001	-	<0.0001	-	0.0006

Supplementary Figure 1 (related to Figures 1 and 2). Single molecule analysis of DNA replication in HMGB1-KO and H1-TKO cells.

(**a**) Immunoblot analysis of HMGB1 levels in primary MEFs obtained from embryos of the indicated genotype. TUBULIN was used as a loading control. Frequency distribution of inter-origin distances (**b**, **g**) and fork rates (**c**, **h**) in MEFs WT, MEFs HMGB1-KO, mES WT and mES H1-TKO. (**d**, **i**) Scatter plot of the distances covered by right-moving and left-moving sister forks during the IdU pulse. The central areas delimited by red lines contain sister forks with less than a 30% length difference. The percentage of asymmetrical signals in each cell type is indicated (lower right of plots).

(**e**, **j**) Statistical analysis of IODs, fork rates and fork asymmetry in the four cell types. (**f**) IGV snapshots showing the SNS coverage at the histone gene locus in mES WT (blue tracks) and in mES H1-TKO (red tracks). The deleted genes H1c, H1d and H1e are highlighted in bold letters.

a

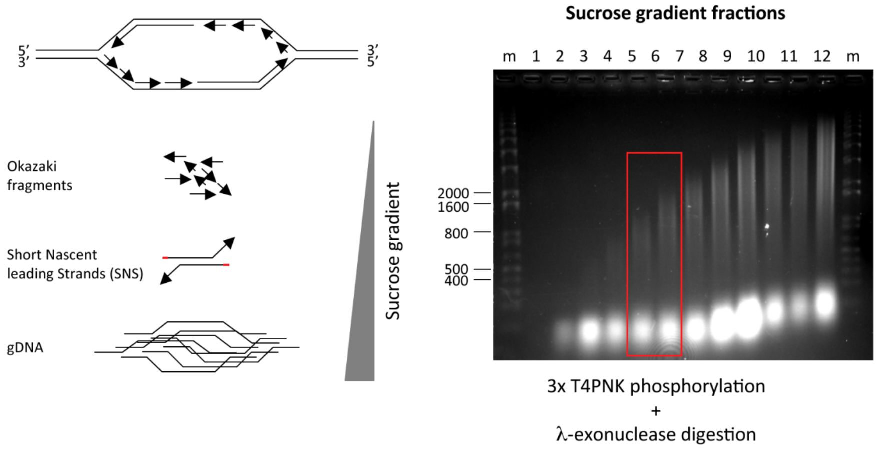

b

SNS sample	Read number	ORI number
MEFs WT-I	55469980	45049
MEFs WT-II	54157881	61020
MEFs HMGB1-I	107801285	65423
MEFs HMGB1-II	119915739	68499
mES WT-I	73122129	94590
mES WT-II	164720257	106891
mES H1-TKO-I	121941851	56615
mES H1-TKO-II	141877552	91273

c

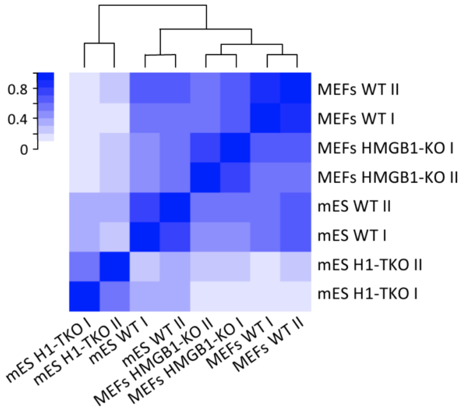

d

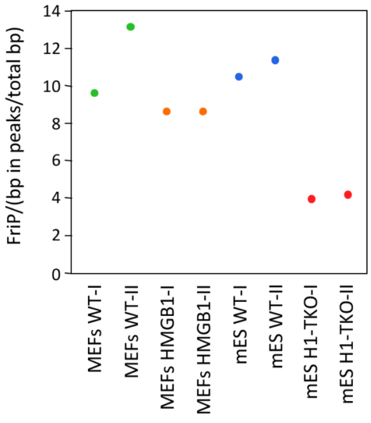

Supplementary Figure 2 (related to Figures 1 and 2). Replication initiation profiling by SNS-Seq.

(**a**) Schematics of sucrose gradient fractionation of replication intermediates and representative gradient profile. The fractions used for SNS library preparations are shown. See Methods for details. (**b**) Summary table of aligned reads and identified ORIs at each SNS-Seq library. Colors are as in Figures 1 and 2. (**c**) Clustered heatmap of pair-wise correlation between ORIs identified at the 8 SNS-Seq experiments illustrated in Figure 2a. (**d**) Fraction of reads in peaks (FriP) analysis (1) in each SNS-Seq library illustrating the low SNS enrichments detected in H1-TKO cells. FriP was calculated

as the number of reads overlapping a peak divided by the total number of reads, normalized by the genome fraction in peaks to account for the differences in ORI numbers between experiments.

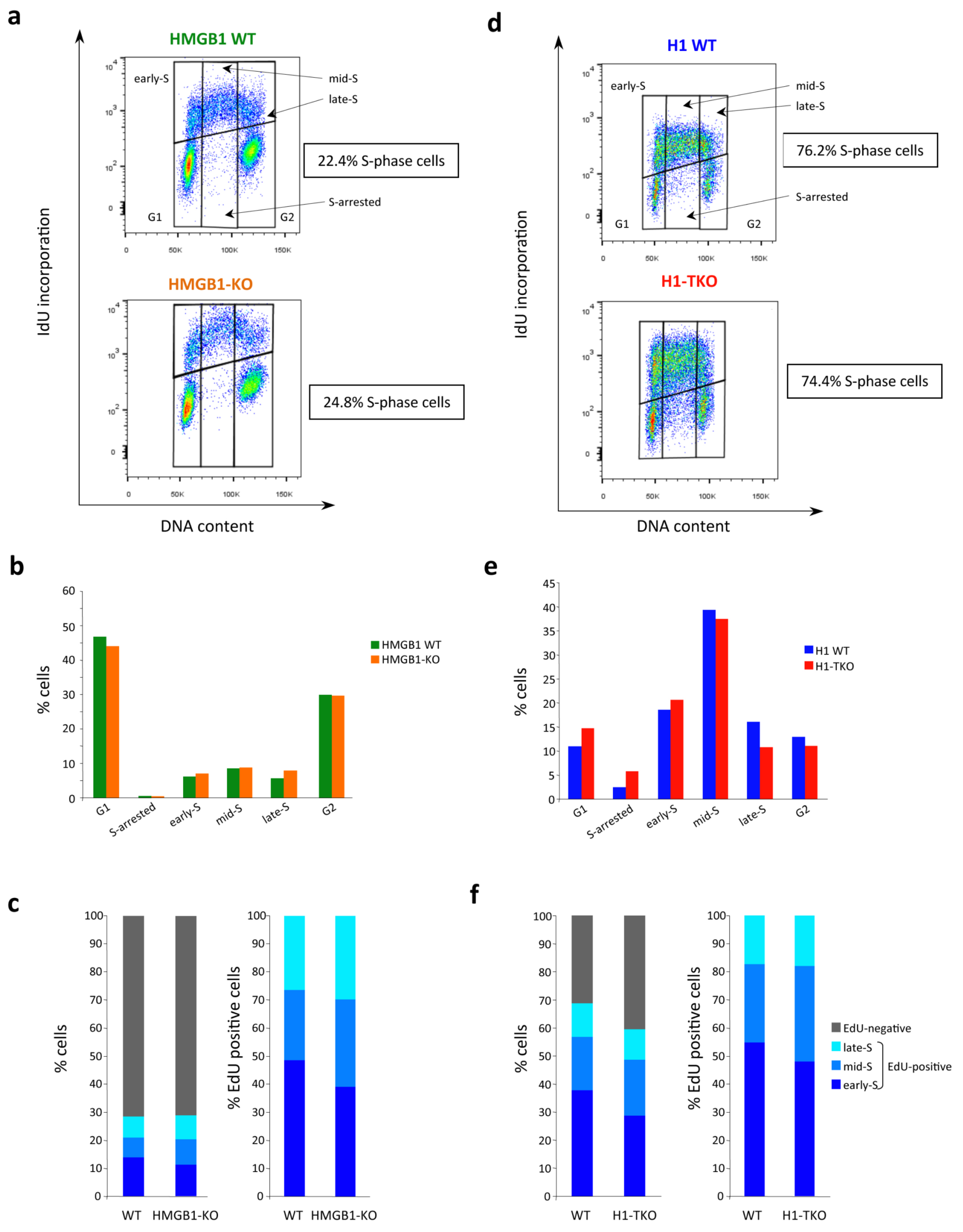

Supplementary Figure 3 (related to Figures 1 and 2). Replication rates of HMGB1-KO and H1-TKO cells.

(**a**, **d**) Cell cycle distribution of MEFs WT, MEFs HMGB1-KO, mES WT and mES H1-TKO. The percentage of actively replicating cells evaluated by IdU incorporation after 20 min pulse is indicated (right side of plots). (**b**, **e**) Percentage of cells at each cell-cycle stage determined from (a). (**c**, **f**) Percentage of early, mid and late-S cells determined by scoring EdU replication-foci patterns (2).

a

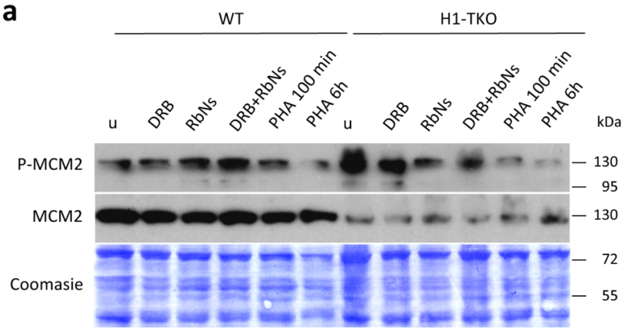

b

Figure 2f	IODs					
	mES WT	mES WT PHA	mES WT Nucleosides	mES H1-TKO	mES H1-TKO PHA	mES H1-TKO Nucleosides
Number of values	92	58	72	96	74	65
Median	71.57	90.81	98.38	52.91	83.26	78.76
Mean	76.63	98.53	113	59.29	92.62	88.22
Std. deviation	32.85	37.94	64.3	26.69	40.65	43.21
P-value	-	< 0.0001	< 0.0001	< 0.0001	< 0.0001	< 0.0001

Figure 2g	Fork rate					
	mES WT	mES WT PHA	mES WT Nucleosides	mES H1-TKO	mES H1-TKO PHA	mES H1-TKO Nucleosides
Number of values	234	268	215	281	333	243
Median	1.137	1.271	1.479	0.7113	1.24	1.381
Mean	1.215	1.314	1.563	0.7512	1.257	1.452
Std. deviation	0.4083	0.4919	0.7604	0.2415	0.3789	0.5303
P-value	-	0.0063	< 0.0001	< 0.0001	< 0.0001	< 0.0001

Figure 2h	Fork assymetry					
	mES WT	mES WT PHA	mES WT Nucleosides	mES H1-TKO	mES H1-TKO PHA	mES H1-TKO Nucleosides
Number of values	42	24	33	55	30	42
Median	16.33	27.61	26.19	35.55	16.16	22.56
Mean	29.64	49.84	41.68	47.01	29.65	30.31
Std. deviation	43.55	63.9	85.56	42.99	41.09	37.17
P-value	-	0.0358	0.3615	0.0012	0.0034	0.0083

c

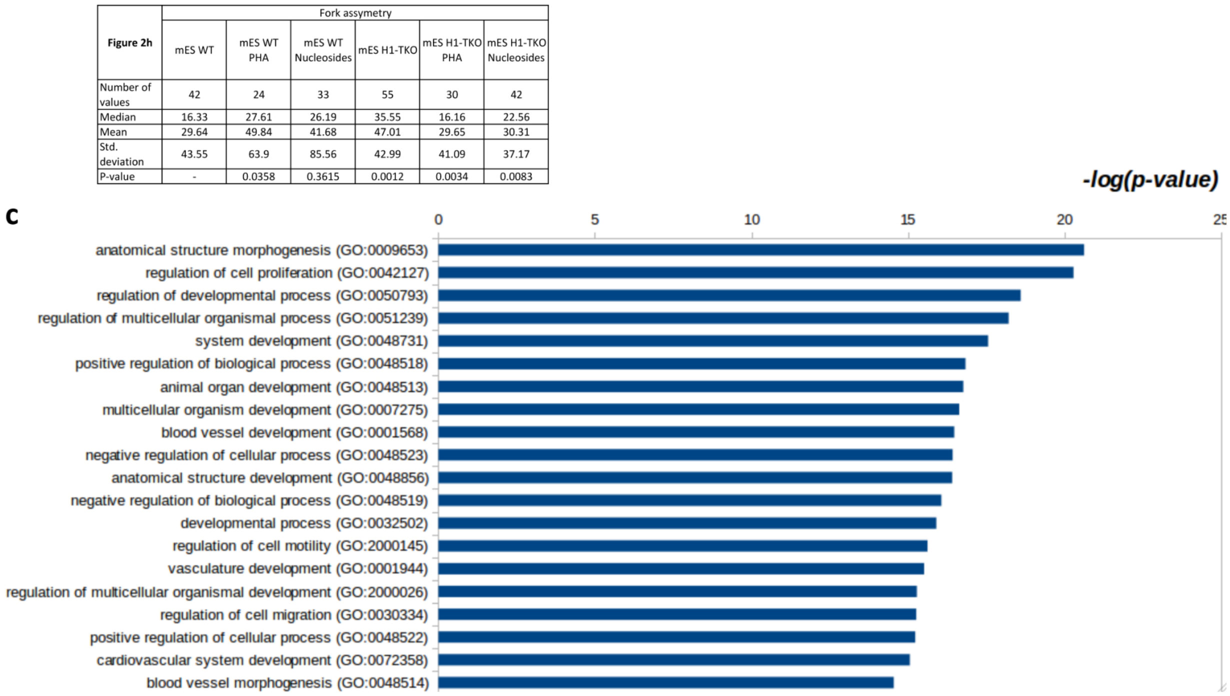

d

GO biological process term	Total	Observed	Expected	Over/Under	Fold Change	P-value
RNA metabolic process (GO:0016070)	2672	42	47,5	-	0,88	1,00E+000
cell cycle (GO:0007049)	1120	17	19,91	-	0,85	1,00E+000
G1/S transition of mitotic cell cycle (GO:0000082)	73	4	1,3	+	3,08	1,00E+000
DNA replication (GO:0006260)	163	0	2,9	-	< 0.2	1,00E+000

Supplementary Figure 4 (related to Figure 2). Replication fork dynamics and gene expression analysis of mES WT and H1-TKO cells.

(**a**) Immunoblot analysis of P-MCM2 and MCM2 levels in WT and H1-TKO cells upon various drug treatments. (**b**) Statistical analysis of IODs, fork rates and fork asymmetry in cells treated with PHA-768491 or ribonucleosides for 100 minutes (Figure 2f-h). (**c**) GO term analysis of genes displaying differential expression between WT and H1-TKO cells. (**d**) Enrichment values of the indicated GO biological processes in differentially expressed genes. Data are from Geeven et al. (2015)[3].

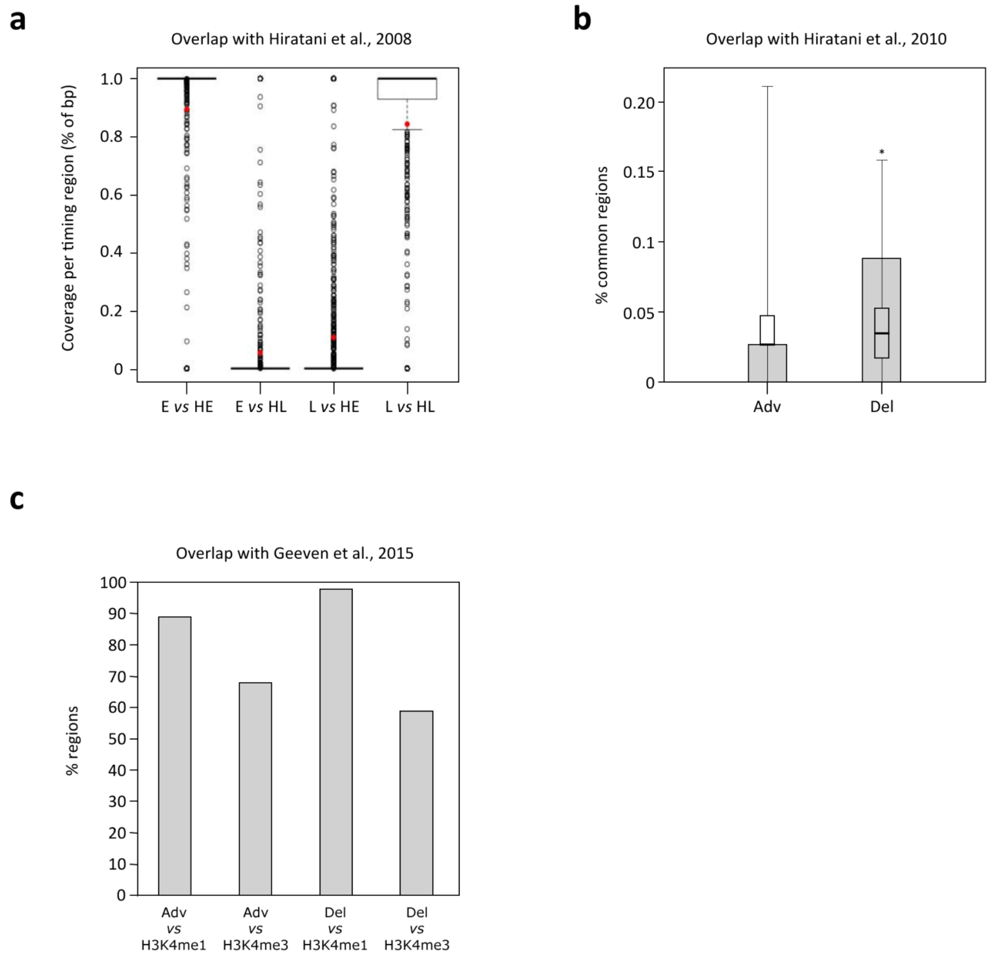

Supplementary Figure 5 (related to Figure 3). Comparative analysis of mES cells replication timing regions with published datasets.

(**a**) Coverage analysis of Early (E) and Late (L) replication domains in WT mES cells with those reported in Hiratani et al. (2008)[4]; HE, Hiratani-Early and HL, Hiratani-Late, in % of bp. Median values are indicated by a black line and means by a red dot. Data not included between the whiskers are plotted as outliers (empty dots). (**b**) Percentage of altered timing regions in H1-TKO cells overlapping with replication domains changing replication timing along mES in vitro differentiation towards neural progenitors (Hiratani et al., 2010)[5]. (**c**) Percentage of regions with advanced (Adv) or delayed (Del) replication timing in H1-TKO cells displaying alterations in H3K4me1 and H3K4me3 levels, as reported for the chromosomal domains with changes in their structural segmentation in the same cell type (Geeven et al., 2015)[3].

a

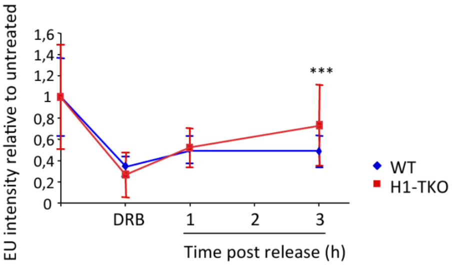

	EU							
	mES WT untreated	mES WT DRB	mES WT 1h-release	mES WT 3h-release	mES H1-TKO untreated	mES H1-TKO DRB	mES H1-TKO 1h-release	mES H1-TKO 3h-release
Number of values	138	114	149	145	100	61	112	103
Median	595.5	213.5	312	292	476.9	117	282.5	342
Mean	619.1	210.2	311.2	297.9	559	146.1	290.4	409.2
Std. deviation	229.8	59.35	80.2	93.18	277.7	120.2	103	213.1
P-value	<0.0001	-	<0.0001	<0.0001	<0.0001	-	<0.0001	<0.0001

b

	S9.6 fluorescence (a.u.)					
**Figure 4d**	mES WT untreated	mES WT RNAse A+	mES WT RNAse H+	mES H1-TKO untreated	mES H1-TKO RNAse A+	mES H1-TKO RNAse H+
Number of values	48	47	47	34	49	51
Median	67.32	60.5	46.68	191.1	142.5	104.3
Mean	71.06	62.29	46.38	237.6	147.6	106.4
Std. deviation	14.37	15.97	6.762	134	25.92	35.27
P-value	-	0.0048	< 0.0001	< 0.0001	< 0.0001	< 0.0001

c

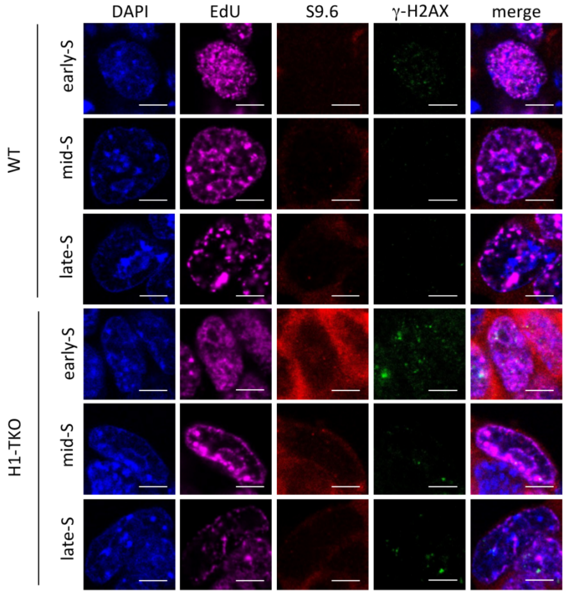

d

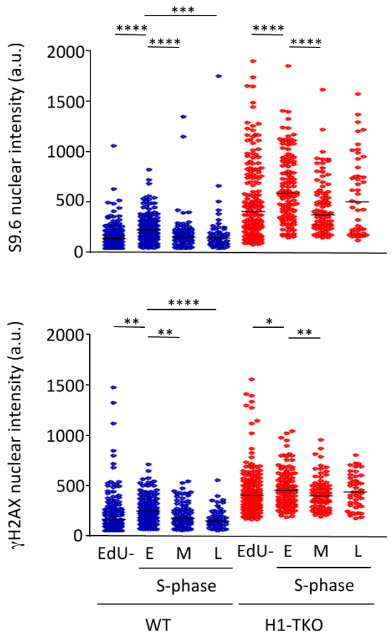

	S9.6 fluorescence (a.u.)							
	mES WT				mES H1-TKO			
	G1/G2/M	Early S	Middle S	Late S	G1/G2/M	Early S	Middle S	Late S
Number of values	141	162	87	54	173	122	86	46
Median	138.199	226.655	157.852	146.215	415.293	600.961	382.093	512.997
Mean	165.378	245.122	190.097	200.311	523.182	673.31	466.136	586.313
Std. deviation	138.445	144.889	187.111	251.063	393.241	378.204	277.752	404.602
P-value	<0.0001	-	<0.0001	0.0003	<0.0001	-	<0.0001	0.1014

	γH2AX fluorescence (a.u.)							
	mES WT				mES H1-TKO			
	G1/G2/M	Early S	Middle S	Late S	G1/G2/M	Early S	Middle S	Late S
Number of values	141	162	87	54	173	122	86	46
Median	152.347	232.102	159.521	133.942	390.979	441.991	386.2	425.187
Mean	222.602	240.198	196.3	161.135	461.666	468.113	403.069	433.364
Std. deviation	225.132	135.53	125.09	99.335	308.094	184.258	160.608	171.578
P-value	0.0012	-	0.0081	<0.0001	0.0309	-	0.0085	0.4062

e

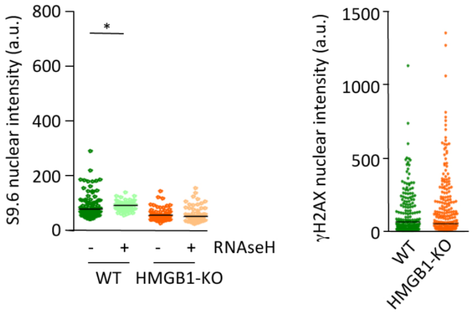

	S9.6 fluorescence (a.u.)				γH2AX fluorescence (a.u.)	
	MEFs WT RNAseH-	MEFs WT RNAseH+	MEFs HMGB1-KO RNAseH-	MEFs HMGB1-KO RNAseH+	MEFs WT	MEFs HMGB1-KO
Number of values	91	88	60	67	189	264
Median	80.54	94.14	57.88	53.22	73.86	62.29
Mean	94.47	92.42	62.37	60.76	136.7	153.8
Std. deviation	43.94	15.88	22.92	27.72	154.7	207.2
P-value	-	0.0226	-	0.2377	-	0.5268

Supplementary Figure 6 (related to Figures 4 and 5). Transcription alterations in H1-TKO cells. **(a)** Nascent transcription recovery upon DRB release in WT and H1-TKO cells (top) and statistical analysis of EU intensity per nucleus (bottom). Values in the graph were normalized to those obtained at untreated samples and the statistical analyses shown were performed at the end point of the analysis. ***P<0.001. (**b**) Statistical analysis of S9.6 intensity per nucleus in both cell types untreated or treated with RNAseA or RNAseH for 36h before immunostaining. (**c**) Representative images of S9.6 and γH2AX immunostaining in early, mid and late-S-phase mES cells. Scale bar, 5 µm. (**d**) Nuclear signal

distribution and statistical analysis of the experiment shown in (c). (**e**) Distribution and statistical analysis of S9.6 (left plots) or γH2AX (right plots) nuclear intensities in WT and HMGB1-KO MEFs.

a

Figure 5a	EU			
	mES WT α-aman-	mES WT α-aman+	mES H1-TKO α-aman-	mES H1-TKO α-aman+
Number of values	87	65	90	88
Median	1272	941.6	932.6	421.5
Mean	1285	959.8	997.2	410.3
Std. deviation	376.8	231	377.4	169.4
P-value	-	<0.0001	-	<0.0001

b

Figure 5b	S9.6 fluorescence (a.u.)					
	mES WT untreated	mES WT α-aman+ RNAse H-	mES WT α-aman- RNAse H+	mES H1-TKO untreated	mES H1-TKO α-aman+ RNAse H-	mES H1-TKO α-aman- RNAse H+
Number of values	182	233	171	175	200	105
Median	217.5	210.7	185.4	572.1	373.4	315.1
Mean	235.9	223.4	210.7	582.3	387.2	327.7
Std. deviation	111.2	107.4	81.19	227.5	148.3	91.46
P-value	-	0.2604	0.0118	-	<0.0001	<0.0001

c

Figure 5c	IODs			
	mES WT α-aman-	mES WT α-aman+	mES H1-TKO α-aman-	mES H1-TKO α-aman+
Number of values	66	57	63	61
Median	88.41	83.19	73.42	76.84
Mean	94.17	86.06	79.49	88.6
Std. deviation	32.01	28.04	37.1	41.32
P-value	-	0.1379	-	0.2672

Figure 5d	Fork rates			
	mES WT α-aman-	mES WT α-aman+	mES H1-TKO α-aman-	mES H1-TKO α-aman+
Number of values	211	232	205	234
Median	1.405	1.395	1.074	1.316
Mean	1.483	1.426	1.13	1.366
Std. deviation	0.6007	0.4129	0.4119	0.4863
P-value	-	0.5298	-	<0.0001

Figure 5e	Fork asymmetry			
	mES WT α-aman-	mES WT α-aman+	mES H1-TKO α-aman-	mES H1-TKO α-aman+
Number of values	39	45	37	45
Median	19.31	14.25	30.83	17.31
Mean	25.01	25.92	58.65	31.55
Std. deviation	20.18	30.63	56.83	43.57
P-value	-	0.3698	-	0.0084

d

Figure 5f	γH2AX fluorescence (a.u.)			
	mES WT α-aman-	mES WT α-aman+	mES H1-TKO α-aman-	mES H1-TKO α-aman+
Number of values	181	233	213	198
Median	237.3	154.9	453.2	252
Mean	266.8	204.1	484.5	313
Std. deviation	121.8	155.5	151.7	182.9
P-value	-	<0.0001	-	<0.0001

e

Figure 6b	IODs							
	mES WT untreated	mES WT DRB	mES WT 1h-release	mES WT 3h-release	mES H1-TKO untreated	mES H1-TKO DRB	mES H1-TKO 1h-release	mES H1-TKO 3h-release
Number of values	43	41	47	50	47	55	48	46
Median	80.72	76.83	74.25	68.03	50.3	81.17	50.77	54.32
Mean	86.14	79.22	79.42	73.31	53.32	81.55	53.23	59.06
Std. deviation	29.76	27.56	31.89	18.44	27.27	27.99	17.48	21.94
P-value	0.2789	-	0.8344	0.451	<0.0001	-	<0.0001	<0.0001

Figure 6c	Fork rate							
	mES WT untreated	mES WT DRB	mES WT 1h-release	mES WT 3h-release	mES H1-TKO untreated	mES H1-TKO DRB	mES H1-TKO 1h-release	mES H1-TKO 3h-release
Number of values	113	114	131	106	194	159	148	171
Median	1.318	1.249	1.175	1.139	0.7327	1.085	0.6314	0.7378
Mean	1.305	1.282	1.247	1.138	0.7711	1.192	0.6847	0.7514
Std. deviation	0.3758	0.3856	0.4087	0.3876	0.3144	0.4695	0.3316	0.3252
P-value	0.614	-	0.2991	0.0087	<0.0001	-	<0.0001	<0.0001

Figure 6d	Fork assymetry							
	mES WT untreated	mES WT DRB	mES WT 1h-release	mES WT 3h-release	mES H1-TKO untreated	mES H1-TKO DRB	mES H1-TKO 1h-release	mES H1-TKO 3h-release
Number of values	22	33	30	25	32	24	29	28
Median	16.53	12.61	20.77	16.4	45.36	18.33	44.05	51.99
Mean	22.77	28.78	26.25	28.45	63.27	20.78	59.71	50.98
Std. deviation	28.87	44.78	22.91	47.19	78.6	16.39	78.27	35.28
P-value	0.8975	-	0.208	0.8016	0.0096	-	0.0188	0.0012

f

Figure 6e	S9.6 fluorescence (a.u.)	
	mES H1-TKO pcDNA	mES H1-TKO pcRNAseH
Number of values	85	102
Median	929.2	403.8
Mean	954.4	462.6
Std. deviation	506.2	172.4
P-value	-	< 0.0001

Figure 6f	γH2AX fluorescence (a.u.)	
	mES H1-TKO pcDNA	mES H1-TKO pcRNAseH
Number of values	85	102
Median	393.8	179.3
Mean	478.1	224.8
Std. deviation	319.3	374.2
P-value	-	< 0.0001

g

Figure 6g-i	IODs		Fork rate		Fork asymmetry	
	mES H1-TKO pcDNA	mES H1-TKO pcRNAseH	mES H1-TKO pcDNA	mES H1-TKO pcRNAseH	mES H1-TKO pcDNA	mES H1-TKO pcRNAseH
Number of values	62	44	286	233	30	29
Median	68.79	71.61	0.7778	0.9508	45.43	20.8
Mean	77.37	73.14	0.8716	0.9958	62.19	35.72
Std. deviation	39.03	34.11	0.3628	0.4749	61.86	30.97
P-value	-	0.7316	-	0.0048	-	0.1314

Supplementary Figure 7 (related to Figures 5 and 6). Recovery of H1-TKO cells replicative stress upon transcription inhibition or R-loop inhibition.

Statistical analysis of nuclear signal intensities of EU (**a**), S9.6 (**b**), and gH2AX (**d**), and IODs, fork rates and fork asymmetry in WT and H1-TKO mES cells untreated or treated with α-amanitin (**c**) or DRB (**e**). Statistical analysis of S9.6 and gH2AX (**f**), and IODs, fork rates and fork asymmetry (**g**) in H1-TKO cells transfected with an empty vector or with a RNAseH1-overexpression vector.

a

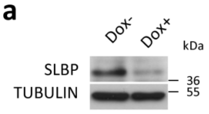

b

Figure 7b-d	IODs		Fork rate		Fork asymmetry	
	HCT-shSLBP Dox-	HCT-shSLBP Dox+	HCT-shSLBP Dox-	HCT-shSLBP Dox+	HCT-shSLBP Dox-	HCT-shSLBP Dox+
Number of values	109	101	505	414	68	61
Median	65.83	63.1	1.003	1.441	12.56	13.44
Mean	75.52	73.04	1.104	1.546	20.3	22.4
Std. deviation	42.14	33.87	0.4474	0.5757	27.13	24.69
P-value	-	0.9991	-	<0.0001	-	0.477

Supplementary Figure 8 (related to Figure 7). Single molecule analysis of DNA replication in HCT-shSLBP cells.

Immunoblot analysis of SLBP levels in control and Doxicyclin-induced SLBP-KD HCT cells. TUBULIN was used as a loading control. (**b**) Statistical analysis of IODs, fork rates and fork asymmetry in the same conditions.

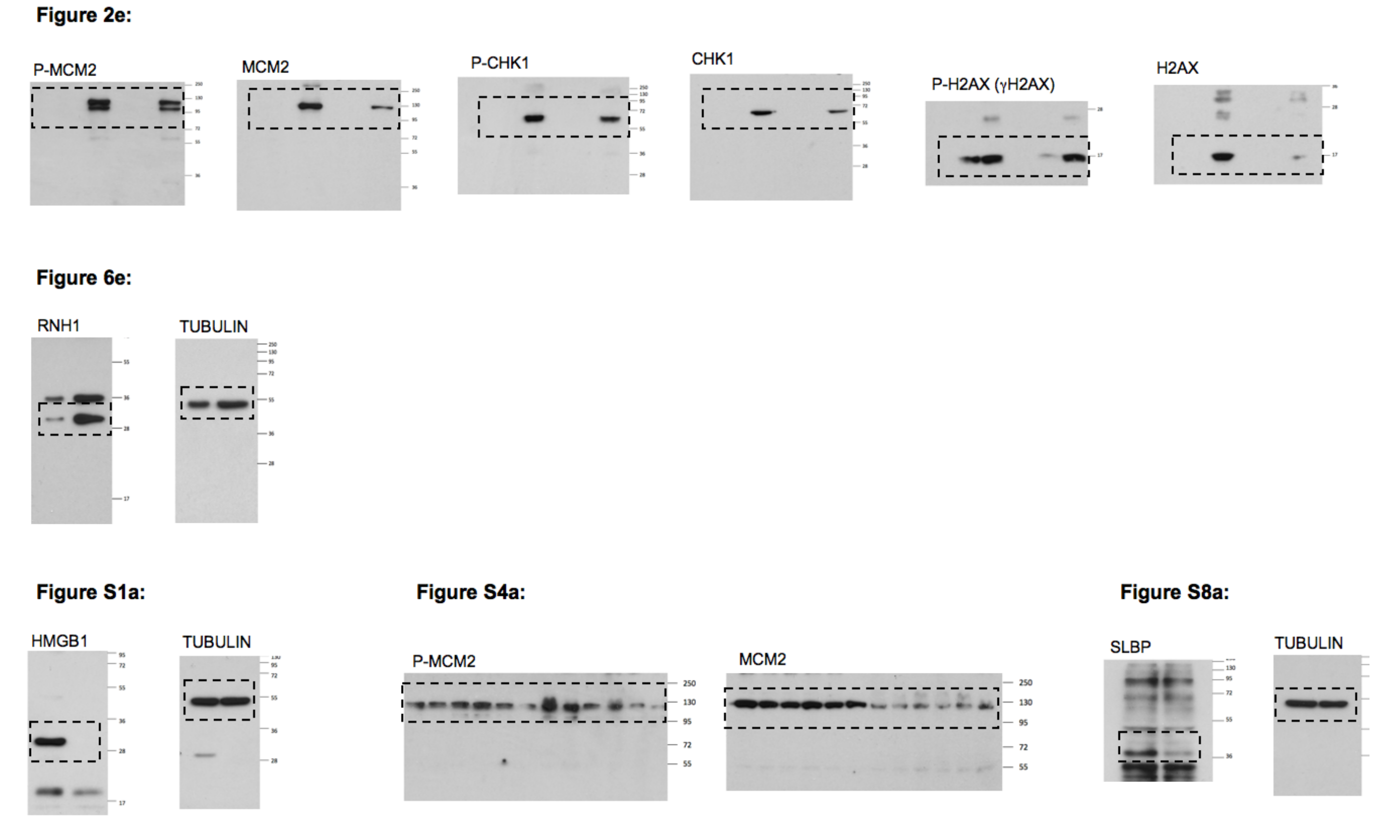

Supplementary Figure 9 (related to Figures 2e, 6e, S1a, S4a and S8a). Full images of WB.

**Primer**	**Sequence (5' to 3')**	**Annealing T^{ra} (ºC)**
Med13l-Ex1F	CTGGAGGATTGTCACTCCAACC	62
Med13l-In1R	TCCGGGAGGAGAAAGTTGCG	
Med13l-Ex4F	TGTGCGGCCCTATGACAAGG	64
Med13l-In4R	CAGATAACAGATACGCCAGCCC	
Med13l-Ex5F	AGTGTGGAGATAGCTCAGCACC	64
Med13l-In5R	TGCACGCAGTTACGCTGGTG	
Med13l-In-lastF	AGGTGGCCATGCTGGTGTGC	64
Med13l-Ex-lastR	CTGGATTGCACGTGAGCCAG	
Inpp5a-Ex1F	ACCGCGGTCCTGCTGGTCAC	64
Inpp5a-In1R	GAAAATGGGGATGTCAGGGTCC	
Inpp5a-Ex4F	AGAATACAACAGGGCGCGTGTC	64
Inpp5a-In4R	GCATGCGTGCCGACTTAGTAC	
Inpp5a-Ex5F	GGAAGCTTTTATTTTCTTCACGAATCC	64
Inpp5a-In5R	GACAACAGAGCTAGAGGGACC	
Meg3F	GACCCCCAGATCACAGAGAA	60
Meg3R	AAAGAACCCTGCCTCCAAAT	
RianF	CCTGGTGAACACATCCCTCT	60
RianR	TTTCCTTTCCCCTTGGACTT	
AirnF	AAAGGGAAGGGAAAGCTCAG	62
AirnR	GCATTAAAACCCTCCGAACC	
Pias3F	TATGGGCTGGATGGTGAGTG	60
Pias3R	GAGACCTGTGGGTGGTTAAG	
AK13F	CATGTTGCCTTCGTCATGGTG	62
AK13R	AGTTATGTCCCCAGCGTGC	

Supplementary Table 1 (related to Figures 4 and 7). **Primers and qPCR conditions.**

Quantitative real-time PCR (qPCR) was performed in an ABI Prism 7900HT instrument (Applied Biosystems) with HotStar Taq polymerase (Qiagen) and SYBR Green (Molecular Probes). Reactions were performed through 15 minutes at 95ºC and 40 cycles of 30 seconds at 95ºC, 30 seconds at the annotated annealing temperature and 1 minute at 72ºC. Conditions for each pair of primers were empirically adjusted to a slope of -3.3±0.3 and R2>0.99 using four serial five-fold dilutions of sonicated genomic DNA. Reactions were performed in duplicate in at least in two independent preparations. Analyses were carried out using the ABI Prism 7900HT SDS Software (version 2.4).

Supplementary References

1. Landt, S. G. et al., ChIP-seq guidelines and practices of the ENCODE and modENCODE consortia. *Genome Res.* **22**, 1813-1831 (2012).

2. Nakamura, H., Morita, T. & Sato, C. Structural organizations of replicon domains during DNA synthetic phase in the mammalian nucleous. *Exp. Cell Res*. **165**, 291-297 (1986).

3. Geeven, G., Zhu, Y., Kim, B. J., Bartholdy, B. A., Yang, S. M., Macfarlan, T. S., Gifford, W. D., Pfaff, S. L., Verstegen, M. J., Pinto, H., Vermunt, M. W., Creyghton, M. P., Wijchers, P. J., Stamatoyannopoulos, J. A., Skoultchi, A. I. & de Laat, W. Local compartment changes and regulatory landscape alterations in histone H1-depleted cells. *Genome Biol*. **16**, 289 (2015).

4. Hiratani. I., Ryba, T., Itoh, M., Yokochi, T., Schwaiger, M., Chang, C.W., Lyou, Y., Townes, T. M., Schübeler, D. & Gilbert, D. M. Global reorganization of replication domains during embryonic stem cell differentiation. *PLoS Biol.* **6**: e245 (2008).

5. Hiratani, I., Ryba, T., Itoh, M., Rathjen, J., Kulik, M., Papp, B., Fussner, E., Bazett-Jones, D. P., Plath, K., Dalton, S., Rathjen, P. D. & Gilbert, D. M. Genome-wide dynamics of replication timing revealed by in vitro models of mouse embryogenesis. *Genome Res.* **20**, 155-69 (2010).
