# Supplementary material for: Chromatin conformation regulates the coordination between DNA replication and transcription
Source: Nat Commun. 2018 Apr 23;9:1590. doi: 10.1038/s41467-018-03539-8 (PMC5913246; doi:10.1038/s41467-018-03539-8)
Supplement: Supplementary file 3 — Description of Additional Supplementary Files [file 41467_2018_3539_MOESM3_ESM.pdf]

Description of Additional Supplementary Files

File Name: Supplementary Data 1
Description: Genomic regions displaying altered replication timing in mES H1-TKO cells.
